# 754. Utilization of Internal Special Program Review to Institute Change in an Academic ID Fellowship

**DOI:** 10.1093/ofid/ofad500.815

**Published:** 2023-11-27

**Authors:** Adrienne Carey, Melissa Pender, Paloma F Cariello

**Affiliations:** University of Utah School of Medicine; University of Utah, Salt Lake City, Utah; University of Utah School of Medicine

## Abstract

**Background:**

Fellowship accreditation is crucial to ensure adequate clinical education for physicians in training, yet there is limited data on use of accreditation survey results for program quality improvement. The Accreditation Council for Graduate Medical Education (ACGME) administers annual surveys to trainees and core faculty to monitor education and identify potential non-compliance with ACGME accreditation standards. Our purpose is to describe the creation of an internal program review process for program improvement based on results of nationally implemented accreditation surveys.

**Methods:**

We collected data from standardized ACGME survey responses administered to core faculty and fellows at our academic Infectious Diseases fellowship program. The identification of areas scoring lower than 20% in comparison with national average prompted creation of a Special Program Review (SPR) committee comprised of a program director, a program coordinator, and a trainee from programs outside of the program under review. This committee met separately with core faculty, trainees, and program leadership to compile an assessment of each survey component and opportunities for improvement. This review process of survey results was completed annually from 2020-2022.

**Results:**

Between 2020-2022, surveys were completed by 100% of fellows (7/7 or 8/8) and up to 92% of core faculty (11/12). Key findings raised concerns involving clinical education, faculty supervision, patient safety, and teamwork. Actions taken in response to the SPR included the creation of: an updated backup and weekend coverage system, new didactic curricula, a morbidity and mortality conference, documentation limits, informal fellow/faculty dinners, opt out counseling, and a division culture committee.

**Conclusion:**

A non-punitive internal review process mimicking accreditation standards is valuable for program assessment and improvement as means to identify areas of risk for accreditation issues at a national level. This review process is generalizable, as all accredited programs complete the survey annually. The quality improvement interventions taken will differ depending on survey findings and areas to be addressed.

**Disclosures:**

**All Authors**: No reported disclosures

